# Sexually Transmitted Infection (STI) Incidence, STI Screening, and Human Immunodeficiency Virus Preexposure Prophylaxis Uptake in United States Veterans With Opioid Use Disorder in Long Island, New York

**DOI:** 10.1093/ofid/ofae429

**Published:** 2024-07-22

**Authors:** Pronoma Srivastava, Viraj Modi, Audun J Lier

**Affiliations:** Department of Internal Medicine, Renaissance School of Medicine at Stony Brook University, Stony Brook, New York, USA; Department of Medicine, Northport Veterans Affairs Medical Center, Northport, New York, USA; Department of Medicine, Northport Veterans Affairs Medical Center, Northport, New York, USA; Division of Infectious Diseases, Department of Medicine, Northport Veterans Affairs Medical Center, Northport, New York, USA

**Keywords:** HIV, injection drug use, opioid use disorder, PrEP, sexually transmitted infections

## Abstract

**Background:**

Opioid use disorder (OUD) confers increased risk of contracting bloodborne and sexually transmitted infections (STIs). Limited data exist on infectious disease screening and preexposure prophylaxis (PrEP) usage among United States Veterans (USVs) with OUD, including persons who inject drugs (PWID). This study aimed to evaluate the epidemiology of human immunodeficiency virus (HIV), hepatitis C virus (HCV), bacterial STIs, and PrEP uptake in USVs with OUD, including PWID.

**Methods:**

A retrospective chart review of USVs with OUD seeking care at Northport Veterans Affairs Medical Center between 2012 and 2022 was completed. Sociodemographics, HIV, HCV, STI testing rates and diagnosis, and PrEP uptake were compared between USVs, stratified by injection drug use history.

**Results:**

We identified 502 USVs with OUD; 43% had a history of injection drug use. Overall, 2.2% of USVs had HIV and 28.7% had HCV. An STI was diagnosed in 10% of USVs, most frequently syphilis (1.8%). PWID were more likely to be tested for HIV (93.5% PWID vs. 73.1% non-PWID; *P* < .001), HCV (95.8% PWID vs. 80.8% non-PWID; *P* < .001), and syphilis (80% PWID vs. 69.2% non-PWID; *P* = .006). Total gonorrhea and chlamydia testing rates were 31.9% and 33.7%, respectively, without difference between the groups. PrEP was prescribed in 1.2% of USVs.

**Conclusions:**

In USVs with OUD, gonorrhea and chlamydia screening occurred less frequently than syphilis, HCV, and HIV. PWID were more likely to be screened for HIV, HCV, and syphilis. PrEP uptake was low. Both PWID and non-PWID may benefit from increased STI screening and linkage to PrEP.

## Teaser

Low rates of bacterial sexually transmitted infection screening and preexposure prophylaxis uptake were observed in United States Veterans with opioid use disorder at our institution, including in Veterans with a history of injection drug use.

## Graphical Abstract

**Graphical Abstract:**
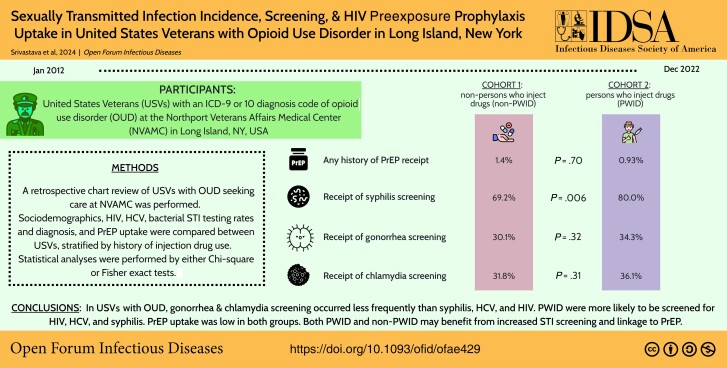
This graphical abstract is also available at Tidbit: https://tidbitapp.io/tidbits/sexually-transmitted-infection-incidence-screening-hiv-pre-exposure-prophylaxis-uptake-in-united-states-veterans-with-opioid-use-disorder-in-long-island-new-yorkcenter-nvamc-in-long-island-ny-usa?utm_campaign=tidbitlinkshare&utm_source=ITP

Approximately 6.1 million persons in the United States (US) have a diagnosis of opioid use disorder (OUD) [1]. In the US military, rising rates of opioid misuse among US Veterans (USVs) between 2010 and 2019 have contributed to a 53% increase in overdose deaths from heroin or fentanyl and its synthetic derivates [2–5]. Misuse of opioids via injection is also associated with increased risk of human immunodeficiency virus (HIV) and hepatitis C virus (HCV) acquisition via shared works, as well as bacterial sexually transmitted infections (STIs) via increased risky sexual behavior associated with substance use [6–11].

HIV preexposure prophylaxis (PrEP) reduces sexual and bloodborne transmission of HIV by 99% and 74%, respectively, and is recommended by the Centers for Disease Control and Prevention (CDC) for persons at risk for HIV, including persons who inject drugs (PWID), those with high-risk sexual practices, or those with recent bacterial STI [12–14]. However, PrEP utilization rates remain low [15]. Furthermore, national incidence of bacterial STIs, especially gonorrhea and syphilis, has skyrocketed [16].

While the Veterans Health Administration (VHA) is the largest provider of substance use disorder (SUD) treatment in the United States, previous studies of STI testing in USVs with OUD noted low testing rates of HIV, gonorrhea, chlamydia, and syphilis [17, 18]. Further, USVs who inject drugs were not included and PrEP uptake was not assessed. Thus, we aimed to investigate rates of bacterial STI (eg, chlamydia, gonorrhea, and syphilis) testing, case positivity, and PrEP uptake in a population of USVs with OUD, including a subset of USVs with a history of injection drug use (IDU). We hypothesized that USVs with OUD and a history of IDU would have higher rates of HIV, HCV, and bacterial STIs and lower PrEP uptake than USVs with OUD without a history of IDU.

## METHODS

### Study Design, Setting, and Population

A retrospective cohort study of all USVs aged 18 years or older with an *International Classification of Diseases*, *9th Revision* (*ICD-9*) or *10th Revision* (*ICD-10*) diagnosis code of OUD, with either an inpatient or outpatient encounter at the Northport Veterans Affairs Medical Center (NVAMC) between 1 January 2012 and 31 December 2022 was performed (Supplementary Table 1.1). This study was approved by the NVAMC institutional board review (approval number 1683516-1).

### Data Collection

The VHA electronic health record (EHR) was utilized for data collection. For data that could not be obtained in the Computerized Patient Record System, the Joint Longitudinal Viewer was used to access additional testing data that had occurred at other VHA locations. Demographic data; housing status; concomitant substance use history; psychiatric comorbidities; history of military sexual trauma (MST); current or past receipt of US Food and Drug Administration–approved medications for opioid use disorder (MOUD), including methadone (full opioid receptor agonist), buprenorphine (partial opioid receptor agonist), and extended-release naltrexone (opioid receptor antagonist); and receipt of hepatitis A virus (HAV) and hepatitis B virus (HBV) vaccines were obtained. Evidence of homelessness was identified by documentation within the EHR. A subsample of USVs with OUD and history of IDU were identified through review of emergency room, outpatient, inpatient, residential treatment, and social work documentation. PWID were defined as having either a past or current documented history of IDU with either synthetic, semi-synthetic, or natural opioids; methamphetamines; and/or cocaine. USVs with OUD and no evidence of IDU were designated as non-PWID and were used as a comparison group for our analysis (Supplementary Table 1.2).

### Outcome Variables and Definitions

The primary outcome of PrEP receipt was defined by receipt of a >30-day course of tenofovir (either tenofovir disoproxil fumarate or tenofovir alafenamide) and emtricitabine or cabotegravir injection in conjunction with provider documentation in the EHR prior to or during calendar year 2022. Secondary outcome data were collected for receipt of any previous HIV or HCV testing as well as case positivity. A positive HIV case was defined as either having a (1) positive screening antibody test with positive confirmatory antibody testing, (2) positive HIV viral load, or (3) documentation of HIV on the EHR problem list. A positive HCV case was defined as either having positive screening antibody test with or without a concurrent positive HCV viral load, or if HCV was documented in the EHR problem list.

Additional secondary outcome data were collected for receipt of bacterial STI testing (chlamydia, gonorrhea, and syphilis), total number of previous tests received per USV, and case positivity. The total number of tests for chlamydia and gonorrhea were counted by distinct patient, specimen collection date, and anatomic source (eg, genitourinary, oropharyngeal, or rectal). A positive test for gonorrhea or chlamydia was defined as a positive nucleic acid amplification test from a genitourinary, oropharyngeal, or rectal specimen. A syphilis case was defined as an individual having either (1) documentation within the EHR of a self-reported history of syphilis or inclusion in the problem list, or (2) positive concurrent non-treponemal and treponemal testing (or positive treponemal test with a positive non-treponemal test). We considered a non-treponemal test as positive if the titer was at least 4-fold greater than the preceding titer or the presence of ≥1:1 titer with initial testing or following a negative test within the EHR (Supplementary Table 1.2).

### Statistical Analysis

Descriptive statistics for birth sex, self-reported race, homelessness, insurance, unemployment history, mental health diagnosis, incarceration history, and receipt of MOUD were compared between PWID and non-PWID via χ^2^ analysis. Differences in receipt of STI testing, case positivity, receipt of HAV and HBV vaccination, and PrEP uptake, stratified by IDU status, were compared via χ^2^ or Fisher exact test using Microsoft Excel. Differences in continuous variables (eg, age) were compared via 2-sample *t* tests. Significance was defined as *P* < .05.

## RESULTS

### Demographics

**Figure 1.**
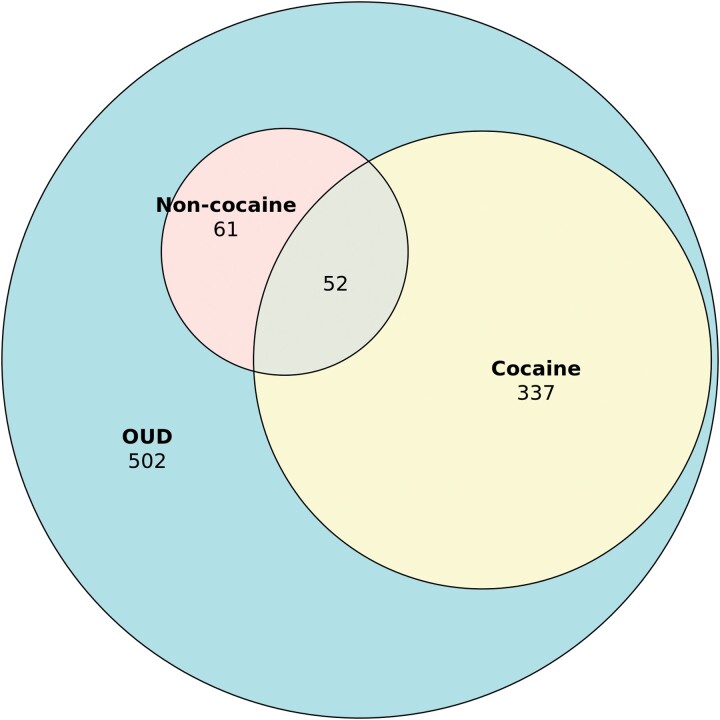
Euler diagram of number of United States Veterans with opioid use disorder (OUD), stratified by stimulant use type.

A total of 502 USVs with a diagnosis of OUD were included (Table 1). Mean age was 52.6 years (standard deviation, 14.0 years), 469 (93.4%) were male, and 396 (78.9%) were White. Two hundred seventy-nine (55.6%) USVs had a history of unemployment, 216 (43%) had a history of uninsured status (notably, services rendered by the VHA does not constitute health insurance), 216 (43%) had a history of homelessness, 216 (43%) had a history of incarceration, and 71 (14.1%) had a history of MST. The 2 most common concomitant substances used were tobacco (n = 357 [71.1%]) and alcohol (n = 352 [70.1%]). A total of 346 (68.9%) USVs had a history of any stimulant use, most commonly cocaine (n = 337 [67.1%]) and non-cocaine stimulants (n = 61 [12.2%]) such as methamphetamine, and 52 (10.4%) USVs had a history of polystimulant use (eg, cocaine and methamphetamine) (Figure 1). A total of 216 (43%) USVs had a history of IDU. The most common comorbid psychiatric diagnoses were posttraumatic stress disorder (n = 275 [54.8%]) and depression (n = 266 [53%]).

**Table 1.** Demographic Characteristics of United States Veterans With Opioid Use Disorder at Northport Veterans Affairs Medical Center, Stratified by Injection Drug Use History

Variable	All Veterans (n = 502)	Non-PWID (n = 286)	PWID (n = 216)	*P* Value
Age, y, mean (SD)	52.6 (14.0)	52.8 (13.2)	52.5 (14.9)	.85
Sex				
Male	469 (93.4)	264 (92.3)	205 (94.9)	.24
Female	33 (6.6)	22 (7.7)	11 (5.1)	
Race				
White	396 (78.9)	228 (79.7)	168 (77.8)	.37
Black	94 (18.7)	50 (17.5)	44 (20.3)	
Asian/Pacific Islander	1 (0.20)	1 (0.35)	0 (0)	
Native American	3 (0.60)	1 (0.35)	2 (0.93)	
Other	8 (1.6)	6 (2.1)	2 (0.93)	
Hispanic ethnicity	28 (5.6)	12 (4.2)	16 (7.4)	.98
History of unemployment	279 (55.6)	141 (49.3)	138 (63.9)	**<**.**001**
History of uninsured status	216 (43.0)	106 (37.1)	110 (50.9)	**.002**
History of homelessness	216 (43.0)	62 (21.7)	154 (71.3)	**<**.**001**
Concomitant substance use				
Alcohol	352 (70.1)	187 (65.4)	165 (76.4)	.**008**
Cocaine stimulant	337 (67.1)	169 (59.1)	168 (77.8)	**<**.**001**
Non-cocaine stimulant	61 (12.2)	25 (8.7)	36 (16.7)	.**007**
Any stimulant use	346 (68.9)	173 (60.5)	173 (80.1)	**<**.**001**
Polystimulant use	52 (10.4)	21 (7.3)	31 (14.4)	.**011**
Marijuana	200 (39.8)	111 (38.8)	89 (41.2)	.59
Tobacco	357 (71.1)	189 (66.1)	168 (77.8)	.**004**
Prescribed MOUD				
Currently on MOUD	222 (44.2)	110 (38.5)	112 (51.9)	.**003**
Previously on MOUD	188 (37.5)	89 (31.1)	99 (45.8)	**<**.**001**
Prescribed naloxone	357 (71.1)	200 (69.9)	157 (72.7)	.50
History of injection substance use	216 (43.0)	0 (0)	216 (100)	
History of incarceration	216 (43.0)	110 (38.5)	106 (49.1)	.**002**
Concomitant psychiatric diagnosis				
PTSD	275 (54.8)	155 (54.2)	120 (55.6)	.76
Depression	266 (53.0)	150 (52.5)	116 (53.7)	.78
Anxiety	47 (9.4)	23 (8.0)	24 (11.1)	.24
Bipolar	83 (16.5)	43 (15.0)	40 (18.5)	.30
History of MST	71 (14.1)	41 (14.3)	30 (13.9)	.89

Data are presented as No. (%) unless otherwise indicated. Statistically significant values appear in **bold** text.

Abbreviations: MST, military sexual trauma; MOUD, medication for opioid use disorder; PTSD, posttraumatic stress disorder; PWID, persons who inject drugs; SD, standard deviation.

When stratified by IDU status, PWID were more likely to have a history of unemployment (63.9% PWID vs. 49.3% non-PWID, *P* < .001), homelessness (71.3% PWID vs. 21.7% non-PWID, *P* < .001), and incarceration (49.1% PWID vs. 38.5% non-PWID, *P* = .002) or uninsured status (50.9% PWID vs. 37.1% non-PWID, *P* = .002) (Table 1). Further, PWID were more likely to use cocaine stimulants (77.8% PWID vs. 59.1% non-PWID, *P* < .001) and non-cocaine stimulants (16.7% PWID vs. 8.7% non-PWID, *P* = .007) and to have a history of polystimulant use (14.4% PWID vs. 7.3% non-PWID, *P* = .011), tobacco use (77.8% PWID vs. 66.1% non-PWID, *P* = .004), or alcohol use (76.4% PWID vs. 65.4% non-PWID, *P* = .008). PWID were also more likely to be currently (51.9% PWID vs. 38.5% non-PWID, *P* = .003) and previously (45.8% PWID vs. 31.1% non-PWID, *P* < .001) prescribed MOUD. There were no differences in concomitant psychiatric diagnoses between PWID and non-PWID.

### PrEP Uptake

**Figure 2.**
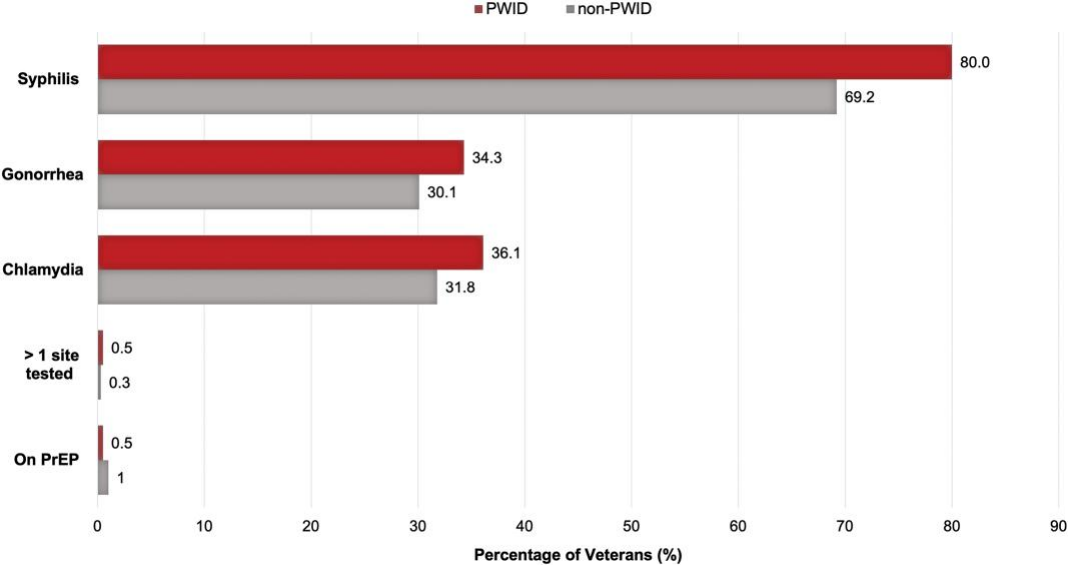
Sexually transmitted infection screening rates and preexposure prophylaxis uptake among US Veterans with opioid use disorder at the Northport Veterans Affairs Medical Center, stratified by injection drug use. Abbreviations: PrEP, human immunodeficiency virus preexposure prophylaxis; PWID, persons who inject drugs.

PrEP was prescribed in 6 (1.2%) USVs (Table 2). There were no differences in current (1.0% PWID vs. 0.5% non-PWID, *P* = .64) or prior (0.5% PWID vs. 0.3% non-PWID, *P* = 1.00) PrEP uptake between PWID and non-PWID (Figure 2). PrEP was prescribed in 5 USVs engaged in condomless sex with multiple partners and in 1 person with IDU.

**Table 2.** Sexually Transmitted Infection (STI) Screening Rates, STI Diagnoses, Vaccination Status, and Human Immunodeficiency Virus Preexposure Prophylaxis Uptake Among US Veterans With Opioid Use Disorder at Northport Veterans Affairs Medical Center, Stratified by Injection Drug Use

Variable	All Veterans (n = 502)	Non-PWID (n = 286)	PWID (n = 216)	*P* Value
HIV				
Screened once	411 (81.9)	209 (73.1)	202 (93.5)	**<**.**001**
Screened more than once	324 (64.5)	156 (54.5)	168 (77.8)	**<**.**001**
Positive	11 (2.2)	2 (0.7)	9 (4.2)	.**008**
HCV				
Screened once	438 (87.3)	231 (80.8)	207 (95.8)	**<**.**001**
Screened more than once	350 (69.7)	172 (60.1)	178 (82.4)	**<**.**001**
Positive	144 (28.7)	10 (3.5)	134 (62.0)	**<**.**001**
Syphilis				
Screened once	371 (73.9)	198 (69.2)	173 (80.0)	.**006**
Screened more than once	197 (39.2)	96 (33.6)	101 (46.8)	.**003**
Positive	9 (1.8)	5 (1.7)	4 (1.9)	1.00
Gonorrhea				
Screened once	160 (31.9)	86 (30.1)	74 (34.3)	.32
Screened more than once	69 (13.7)	38 (13.3)	31 (14.4)	.73
Positive	8 (1.6)	4 (1.4)	4 (1.9)	.73
Chlamydia				
Screened once	169 (33.7)	91 (31.8)	78 (36.1)	.31
Screened more than once	80 (15.9)	41 (14.3)	39 (18.1)	.26
Positive	9 (1.8)	6 (2.1)	3 (1.4)	.74
HPV	4 (0.8)	3 (1.0)	1 (0.5)	.64
*Trichomonas*	1 (0.2)	1 (0.3)	0 (0.0)	1.00
Any previous STI	51 (10.1)	27 (9.4)	24 (11.1)	.54
>1 STI	11 (2.2)	5 (1.7)	6 (2.8)	.54
STI screened, multiple sites	2 (0.4)	1 (0.3)	1 (0.5)	1.00
Vaccination status				
HAV	285 (56.8)	126 (44.1)	159 (73.6)	**<**.**001**
HBV	297 (63.7)	151 (54.3)	146 (77.7)	**<**.**001**
PrEP				
Currently prescribed	4 (0.8)	3 (1.0)	1 (0.5)	.64
Previously prescribed	2 (0.4)	1 (0.3)	1 (0.5)	1.00
Any history of PrEP receipt	6 (1.2)	4 (1.4)	2 (0.93)	.70

Data are presented as No. (%) unless otherwise indicated. Statistically significant values appear in **bold** text.

Abbreviations: HAV, hepatitis A virus; HBV, hepatitis B virus; HCV, hepatitis C virus; HIV, human immunodeficiency virus; HPV, human papillomavirus; PrEP, human immunodeficiency virus preexposure prophylaxis; PWID, persons who inject drugs; STI, bacterial sexually transmitted infection.

### HIV and HCV Testing and Case Positivity

A total of 411 (81.9%) USVs received testing for HIV and 438 (87.3%) for HCV, of which 11 (2.2%) USVs (9 of 11 were PWID) had documented HIV and 144 (28.7%) had documented HCV (134 of 144 were PWID). PWID were more likely to have received testing for HIV (93.5% PWID vs. 73.1% non-PWID, *P* < .001) and HCV (95.8% PWID vs. 80.8% non-PWID, *P* < .001). Among PWID, 168 (77.8%) and 178 (82.4%) USVs received testing more than once for HIV and HCV, respectively (Table 2).

### Bacterial STI Testing and Case Positivity

A total of 371 (73.9%) USVs received testing for syphilis, 160 (31.9%) for gonorrhea, and 169 (33.7%) for chlamydia. Only 2 (0.4%) USVs received testing at >1 anatomic site. PWID were more likely to receive testing for syphilis (80.0% PWID vs. 69.2% non-PWID, *P* = .006) and to receive syphilis testing more than once (46.8% PWID vs. 33.6% non-PWID, *P* = .003) (Figure 2). Testing rates for both gonorrhea (n = 160 [31.9%]) and chlamydia (n = 169 [33.7%]) were low in the entire cohort and there was no difference between the groups. An STI was diagnosed in 51 (10%) USVs, most frequently syphilis (1.8%). There was no difference in any STI case positivity between PWID and non-PWID (Table 2).

### Immunization Status

A total of 285 (56.8%) USVs had received HAV and 297 (63.7%) HBV immunization (Table 2). PWID were more likely to be immunized against HAV (73.6% PWID vs. 44.1% non-PWID, *P* < .001) and HBV (77.7% PWID vs. 54.3% non-PWID, *P* < .001).

## DISCUSSION

In this analysis of USVs with OUD, we identified a middle-aged, predominately male, White, and non-Hispanic population at elevated risk for HIV, HCV, and bacterial STIs. This population had a high frequency of comorbid psychiatric and SUD diagnoses, and a history of sexual violence, homelessness, incarceration, and uninsured status. These factors are known barriers to healthcare access and enhance risk of acquiring HIV, viral hepatitis, and bacterial STIs [11, 19–21]. Moreover, these factors disproportionately affect USVs, a population that has been historically stigmatized [22–24].

To our knowledge, this is the first study to analyze a cohort of USVs with OUD and a history of IDU. Our cohort was identified via manual review of EHR documentation, thus positively confirming IDU status. PWID have previously been difficult to identify in epidemiological analyses, often due to either lack of a corresponding *ICD* code or detailed information regarding IDU in administrative health data sets [25, 26]. As such, previous studies of infectious diseases outcomes in PWID have been limited to use of *ICD* codes that correspond to likely IDU [25–27]. We found that this cohort was more likely to have a history of unemployment, homelessness, polysubstance use (including psychostimulants), and incarceration than USVs with OUD and no history of IDU. PWID have been noted to have greater disparities in social determinants of health and are more likely to have poor HIV outcomes and unmet needs for healthcare services [28]. Our study thus highlights an important USV population that would benefit from wraparound care services, including case management, transportation, housing, mental health, and primary care [29].

Receipt of HIV testing was 81.9% in our cohort, including 93.5% among PWID, which is higher than previously reported in USVs with OUD [18]. This may reflect increased awareness of HIV testing among NVAMC staff (and local Veterans Affairs [VA] locations in New York, New Jersey, and Connecticut, where this cohort frequently obtained healthcare) and/or improved access to HIV testing among USVs with OUD at these VHA locations. Our HIV case positivity was 2.2%, including 4.2% among PWID, which was higher than national rates of HIV in USVs with OUD; however, previous studies in non-Veteran populations with OUD have found a prevalence as high as 8.5% [18, 30]. High HIV screening rates in USVs align with the strategic aims of the US Department of Health and Human Services initiative Ending the HIV Epidemic in the US (EHE), which intends to reduce new HIV infections while increasing HIV screening, treatment, prevention, and linkage to care [31].

We also found a high HCV testing rate of 87.3% among USVs with OUD, including 95.8% among PWID. To our knowledge, this is the first study to look at HCV testing rates among USVs with a history of OUD, including USVs with IDU. Since implementation of a comprehensive National Hepatitis C Program in 1998, the VHA has made tremendous efforts to improve USV HCV testing, linkage to care, and treatment, with previous estimates as high as 80% of the baby boomer birth cohort tested as of fiscal year 2017 [32]. As a result of these policies, rates of HCV testing at the NVAMC and within the national VHA population continue to be significantly higher than HCV screening rates in non-VHA cohorts with OUD, which have ranged from 18.1% to 32.4% [33, 34]. Among PWID, we found an HCV seroprevalence of 62%, which is consistent with previous studies of HCV prevalence in PWID, and underscores the importance of testing, linkage to HCV care, and treatment in this population to reduce risk of HCV transmission to peer injection partners (eg, treatment as prevention) and risk of downstream sequelae of cirrhosis and hepatocellular carcinoma [35–37]. We also noted that 82.4% of USVs with OUD and a history of IDU received HCV testing more than once, which aligns with the US Preventive Services Task Force recommendation of periodic testing for persons with risk factors, including IDU [38].

Syphilis testing rates were also high in our cohort, and we identified a 1.8% case positivity rate that exceeds national rates in USVs with OUD (estimated at 0.66%) [18]. Importantly, testing for gonorrhea and chlamydia were low across the cohort, which supports data previously obtained from a national VHA cohort that noted gonorrhea and chlamydia testing rates as low as 6.1% in USVs with OUD [18]. While gonorrhea and chlamydia infections are both associated with increased risk for HIV acquisition and are nationally reportable diseases, testing remains significantly lower than that for syphilis [39–41]. However, unlike with HIV and HCV infections, bacterial STIs have not been afforded the same recommendation of universal screening by the CDC [42, 43]. Efforts to increase screening for gonorrhea and chlamydia, particularly at extragenital sites that may be asymptomatic, are needed in USVs. This is vital as early identification and treatment of these infections may reduce negative downstream sequelae, as well as acquisition or transmission of HIV [44].

Despite surpassing national rates of PrEP uptake among PWID, PrEP receipt was overall low in our cohort, highlighting an area that warrants increased attention at the federal and regional levels [45]. Explanations for low PrEP uptake and low chlamydia and gonorrhea testing are likely attributable to various factors at the individual, provider, and structural levels as well as biopsychosocial and environmental determinants. Many USVs, particularly sexual, ethnic, and racial minorities, perceive or anticipate bias during healthcare encounters, precluding them from seeking preventive healthcare services or disclosing sexual histories and behaviors that may confer increased risk of STIs, HIV, viral hepatitis, and SUD [3, 46, 47]. Moreover, lack of provider familiarity or comfort with bacterial STI testing, treatment, and preventive counseling, coupled with lack of knowledge about criteria for PrEP receipt, are obstacles that may have contributed to lower rates of gonorrhea or chlamydia testing and PrEP receipt in our cohort. Healthcare providers within the VHA have cited apprehension in eliciting sexual histories and were less likely to initiate discussions about PrEP than their own patients [48–50]. Failure to evoke sexual and substance use histories during patient encounters are missed opportunities to screen for illicit substance use, SUD, and PrEP candidacy or to assess for viral hepatitis and bacterial STI. Culturally sensitive targeted interventions, such as workshops employing interactive role-play for providers to elicit sexual histories, and didactics to educate providers on indications of PrEP prescription, bacterial STI screening, diagnostic testing, treatment, and preventive counseling are needed. Interestingly, the observed higher rates of syphilis testing are likely due to ease of serologic testing, while chlamydia and gonorrhea require swabbed samples from anatomic sites.

Our analysis underscores 3 additional areas of concern. First, in our cohort, USVs with OUD and no history of IDU were less likely to be tested for HCV, HIV, and syphilis or to be vaccinated against HAV or HBV when compared to USVs with a history of IDU. Current CDC guidelines recommending repeat HIV and hepatitis screening among PWID may partially explain this incongruence [38, 51–52]. Given that previous studies demonstrate that persons who use drugs (PWUD) but who do not inject are still at risk for acquiring and transmitting HIV, viral hepatitis, and bacterial STIs, periodic screening for these infections should be offered in this cohort [53–57]. As outbreaks of HAV have also been reported in PWUD without a history of IDU, HAV and HBV vaccination should be equally prioritized in all USVs with OUD [58]. Second, while both cohorts had a higher frequency of MOUD initiation than previously reported among USV populations [59], a sizable number of USVs with OUD were never prescribed MOUD. Moreover, non-PWID with OUD were less likely to receive MOUD than those with IDU. As MOUD is associated with reduced sex risk and injection-related risk behaviors and improved adherence to treatment regimens such as antiretroviral therapy in persons with HIV and direct-acting antiviral therapy in persons with HCV, the provision of MOUD aligns with EHE and its goals of treatment as prevention. Thus, MOUD should be offered to all PWUD with OUD, irrespective of IDU [60–64]. Our findings may therefore reflect barriers at the individual (eg, decreased understanding or self-perceived benefit of MOUD in USVs) or provider level at NVAMC. Further study of this association at other VHA locations should be undertaken. Third, the burden of comorbid polysubstance use disorders in our cohort is substantial, corroborating prior research in USVs that have associated OUD with 1 or more comorbid SUDs [24, 65]. Stimulant use disorder was observed in more than two-thirds of all individuals with OUD in our cohort, mirroring the rising incidence of illicit stimulant use and stimulant-related drug overdoses as well as new HIV epidemics associated with stimulant use in the US [66, 67]. Stimulant use is associated with numerous high-risk sexual behaviors, including condomless sex, transactional sex, and multiple sex partners [52, 68]. Fortunately, provision of PrEP is highly effective in preventing acquisition of HIV among stimulant users with multiple condomless sexual partners [69]. Further research is needed to improve PrEP uptake in USVs who use drugs, including stimulants.

Last, despite the VHA being the largest healthcare system in the US, there is still service fragmentation as SUD clinics do not routinely offer HIV, HCV, bacterial STI testing, or PrEP, and primary care or infectious disease clinics do not routinely offer SUD care in combination with infectious diseases testing or PrEP [70]. These care silos are a barrier to delivering comprehensive care, especially considering the shared biopsychosocial risk factors and bidirectional interplay between SUD and infectious diseases. An integrated, low-barrier model of healthcare delivery for both SUD and infectious diseases screening, prevention (PrEP), and treatment is needed to address these disparities. Alongside this integrated care model should be consideration for provision of long-acting PrEP and long-acting MOUD for PWUD with OUD in order to reduce the barrier of multiple appointment attendance. Fortunately, the VHA has made efforts to address this disparity with creation of harm reduction and syringe services programs. Beginning at the Danville VA (Illinois) in 2017 and expanding to 9 other VHA locations with 12 others in progress, these programs offer access to sterile syringes, fentanyl test strips, naloxone, infectious diseases screening, and linkage to mental health treatment [71]. Hopefully, with expansion of these programs to other VHA locations, integrated infectious diseases and SUD care can become available to all USVs.

Several limitations should be considered when interpreting our data. First, our study evaluated USVs with OUD at a single VA medical center (Northport, New York, affiliated with Stony Brook University) and serves a large suburban population in Long Island. Thus, our findings are of limited generalizability to rural and urban VA facilities, which may have different patient characteristics, population densities, or available resources. Second, the retrospective cohort design may have led to selection bias, including the identification of USVs with OUD who inject drugs, as we were reliant on accurate provider documentation. Thus, the true number of USVs with an IDU history may have been underreported. Moreover, our study design did not include sexual risk profiles, perhaps limiting the identification of USVs eligible for infectious diseases screening and PrEP provision. Further, it was difficult to identify whether a USV had active IDU or remote IDU based on chart review, which we did not further delineate. In addition, some USVs may have declined HIV, viral hepatitis, and bacterial STI testing, overestimating the true number of USVs who were not offered infectious diseases testing during healthcare encounters. Understanding reasons behind a decline in testing receipt is crucial. The retrospective study was also limited to visits within the VHA system and did not account for USVs concurrently seeking healthcare outside the VHA system. Thus, our data analysis may have undercounted the rates of MOUD receipt, infectious diseases testing, diagnosis, and PrEP utilization by USVs with OUD. Finally, our reliance on specific *ICD* codes to capture OUD may have limited sensitivity and led to an underestimate of true OUD prevalence.

## CONCLUSIONS

USVs with OUD are at an increased risk of acquiring HIV, HCV, and bacterial STIs. While testing rates for HIV and HCV remain high overall in our population, there are notable differences in care, especially the significantly lower receipt of infectious diseases testing in USVs with OUD without a history of IDU. Nevertheless, all USVs with OUD would benefit from improved STI screening and provision of PrEP through integrated OUD and infectious diseases care, regardless of IDU status.

## Supplementary Data

Supplementary materials are available at *Open Forum Infectious Diseases* online. Consisting of data provided by the authors to benefit the reader, the posted materials are not copyedited and are the sole responsibility of the authors, so questions or comments should be addressed to the corresponding author.

## Supplementary Material

ofae429_Supplementary_Data

## Notes

***Author contributions.*** P. S. was involved with data collection, data analysis, and manuscript writing and editing. V. M. was involved with data collection and manuscript writing and editing. A. J. L. was involved with study funding acquisition, concept of the manuscript, and manuscript writing and editing.

***Disclaimer.*** The funders were not involved in the research design, analysis or interpretation of the data, or the decision to publish the manuscript.

***Financial support.*** This work was supported by a pilot grant from the Research Corporation of Long Island to author A. J. L.
