# Supplementary material for: Sexually Transmitted Infection (STI) Incidence, STI Screening, and Human Immunodeficiency Virus Preexposure Prophylaxis Uptake in United States Veterans With Opioid Use Disorder in Long Island, New York
Source: Open Forum Infect Dis. 2024 Jul 22;11(8):ofae429. doi: 10.1093/ofid/ofae429 (PMC11289836; doi:10.1093/ofid/ofae429)
Supplement: ofae429_Supplementary_Data [file ofae429_supplementary_data.docx]

**SUPPLEMENTARY MATERIALS*:***

***Table 1.1****. Opioid use disorder diagnosis codes*

| **ICD** | **Diagnosis** | **Codes** |
| --- | --- | --- |
| ICD9 | Opioid-type dependence  Opioid abuse  Nondependent opioid abuse | 304.0x  305.5x |
| ICD10 | Opioid abuse | F11.x |

***Table 1.2.*** *Definitions*

| **Variable** | **Definition** |
| --- | --- |
| Injection drug use (IDU) | Injection drug use and/or persons who inject drugs were identified with the following manual keyword searches within individual patient VA electronic health records:   1. “IVDU”; **OR** 2. “IDU”; **OR** 3. “IV drug use”; **OR** 4. “IV heroin”; **OR** 5. “IV fentanyl”; **OR** 6. “IV cocaine”; **OR** 7. “IV methamphetamine”; **OR** 8. “Intravenous drug use”; **OR** 9. “Intravenous heroin”; **OR** 10. “Intravenous fentanyl”; **OR** 11. “Intravenous methamphetamine”; **OR** 12. “Intravenous cocaine”; **OR** 13. Additional text evidence of “injection” or “IVDU” or “IDU”. |
| History of uninsured status | Lack of private and public (e.g., Medicare, Medicaid) health insurance at any point in time. |
| History of unemployment | Any history of documented unemployment. |
| Homelessness | Individuals were defined as experiencing housing insecurity if they met any of the following criteria prior to or through CY 2022:   1. Veterans currently living in a VA funded community-based residential homeless program; **OR** 2. Those Veterans who have screened positive to the Homeless Screening Clinical Reminder; **OR** 3. Self-reported unstable housing documented by a social worker or healthcare provider in the individual’s electronic health record. |
| Substance use disorder (SUD) | Individuals were defined as having a substance use disorder prior to or through CY 2022 based on having at least one of the following criteria:   1. A corresponding ICD-9 and ICD-10 diagnosis code of alcohol use disorder, cocaine use disorder, tobacco/nicotine use disorder, marijuana use disorder and/or other stimulant use disorder (methamphetamine); **OR** 2. Alcohol use disorder, cocaine use disorder, tobacco/nicotine use disorder, marijuana/cannabis use disorder and/ or other stimulant use disorder (methamphetamine) included on their problem list. |
| Medications for opioid use disorder (MOUD) | Individuals were considered to have received medications for opioid use disorder (MOUD) if they had been prescribed any of the following medications for >30-day duration documented within the VHA administrative data prior to or through CY 2022:   1. Buprenorphine (and extended-release buprenorphine) 2. Methadone 3. Extended-release Naltrexone |
| Incarceration history | Self-reported or documented history of incarceration documented prior to or through CY 2022. |
| Mental health diagnosis | Individuals were defined as having a diagnosis of anxiety disorder, bipolar disorder, depressive disorder, post-traumatic stress disorder, schizophrenia, or other mental health disorder prior to or through CY 2022 based on either of the following criteria:   1. ICD-9 and ICD-10 codes; **OR** 2. Any of the above included on patient problem list; **OR** 3. Psychologic/psychiatric encounter documentation in the individual’s electronic health record. |
| Military sexual trauma (MST) | Self-reported MST documented prior to or through CY 2022. |
| Human Immunodeficiency Virus (HIV) screening | Individuals were defined as having received HIV testing if they met any of the following criteria in VHA administrative data prior to or through CY 2022:   1. HIV antibody combined immunoassay; **OR** 2. HIV antibody. |
| HIV diagnosis | Individuals were defined as having a diagnosis of HIV if they met any of the following criteria in VHA administrative data prior to or through CY 2022:   1. Positive HIV antibody combined with positive confirmatory testing; **OR** 2. Positive HIV viral load; **OR** 3. Prescribed an HIV antiretroviral medication for ≥31 continuous days within the VHA; **OR** 4. HIV included on their problem list. |
| Hepatitis C virus (HCV) screening | Individuals were defined as having been screened for HCV if they had any of the following laboratory results documented in VHA administrative data prior to or through CY 2022:   1. HCV antibody immunoassay; **OR** 2. HCV RNA nucleic acid amplification testing (NAAT). |
| HCV diagnosis | Individuals were defined as having a diagnosis of HCV infection if they met any of the following criteria in VHA administrative data prior to or through CY 2022:   1. Positive HCV antibody immunoassay; **OR** 2. Positive HCV RNA viral load; **OR** 3. Hepatitis C infection included on problem list. |
| Syphilis screening | Individuals were defined as having been screened for Syphilis if they had any of the following laboratory results documented in VHA administrative data prior to or through CY 2022:   1. Nontreponemal serologic testing (e.g., RPR, VDRL); **OR** 2. Treponemal serologic testing (e.g., FTA-ABS, TP-PA, EIA); **OR** 3. *Treponema pallidum* NAAT. |
| Syphilis diagnosis | Individuals were defined having a diagnosis of Syphilis if they met any of the following criteria in VHA administrative data prior to or through CY 2022:   - 1. Positive nontreponemal (e.g., RPR, VDRL) combined with positive treponemal serologic testing (e.g., FTA-ABS, TP-PA, EIA); **OR**   2. Positive treponemal serologic testing (e.g., FTA-ABS, TP-PA, EIA) combined with positive nontreponemal (e.g., RPR, VDRL); **OR**   3. 4-fold increase in nontreponemal (e.g., RPR, VDRL) serologic titer; **OR**   4. Self-reported history of primary syphilis, secondary syphilis, tertiary syphilis, or neurosyphilis documented in individual electronic health record; **OR**   5. Syphilis included on their problem list. |
| Chlamydia screening and diagnosis | Individuals were defined as having been screened for *Chlamydia trachomatis* if they had any of the following laboratory results documented in VHA administrative data prior to or through CY 2022:   1. Genitourinary (e.g., urine, urethral swab) NAAT; **OR** 2. Anal (e.g., rectal swab) NAAT; **OR** 3. Oropharyngeal (e.g., oropharyngeal swab) NAAT; **OR** 4. Conjunctival (e.g.) NAAT.   Individuals were defined as having a diagnosis of *Chlamydia trachomatis* if they met any of the following criteria documented in VHA administrative data prior to or through CY 2022:   1. Positive NAAT from genitourinary, anal, oropharyngeal, or conjunctival specimens |
| Gonorrhea screening and diagnosis | Individuals were defined as having been screened for *Neisseria gonorrheae* if they had any of the following laboratory results documented in VHA administrative data prior to or through CY 2022:   1. Genitourinary (e.g., urine, urethral swab) NAAT; **OR** 2. Anal (e.g., rectal swab) NAAT; **OR** 3. Oropharyngeal (e.g., oropharyngeal swab) NAAT   Individuals were defined as having a diagnosis of *Neisseria gonorrheae* if they met any of the following criteria documented in VHA administrative data prior to or through CY 2022:   1. Positive NAAT from genitourinary, anal, or oropharyngeal specimens |
| Human Papillomavirus (HPV) diagnosis | Individuals were defined as having a diagnosis of HPV infection if they met any of the following criteria documented in VHA administrative data prior to or through CY 2022:   1. Positive Pap smear; **OR** 2. Positive HPV PCR of genitourinary or oropharyngeal anatomic sites. |
| Trichomonas diagnosis | Individuals were defined as having a diagnosis of *Trichomonas vaginalis* infection if they met any of the following criteria documented in VHA administrative data prior to or through CY 2022:   1. Positive *T. vaginalis* rapid antigen testing of vaginal discharge; **OR** 2. Positive wet mount microscopy (motile trichomonads visualized) of vaginal discharge; **OR** 3. Documented self-report of recent *T. vaginalis* infection in a sexual partner; **OR** 4. Positive *T. vaginalis* NAAT of urethral or vaginal discharge. |
| HIV Pre-Exposure Prophylaxis (PrEP) | Individuals were considered to have received pre-exposure prophylaxis to prevent HIV infection if they had met the following criteria in VHA administrative data prior to or through CY 2022:   1. Prescription of tenofovir (disoproxil fumarate or alafenamide) with emtricitabine for >30 days; **AND** 2. Negative HIV antibody combined testing; **OR** 3. Negative HIV viral load. |
